# Calcium Regulation on the Atrial Regional Difference of Collagen Production Activity in Atrial Fibrogenesis

**DOI:** 10.3390/biomedicines9060686

**Published:** 2021-06-17

**Authors:** Cheng-Chih Chung, Yung-Kuo Lin, Yao-Chang Chen, Yu-Hsun Kao, Yung-Hsin Yeh, Yi-Jen Chen

**Affiliations:** 1 Division of Cardiology, Department of Internal Medicine, School of Medicine, College of Medicine, Taipei Medical University, Taipei 11031, Taiwan; michaelchung110@gmail.com (C.-C.C.); yklin213@yahoo.com.tw (Y.-K.L.); 2 Division of Cardiovascular Medicine, Department of Internal Medicine, Wan Fang Hospital, Taipei Medical University, Taipei 11696, Taiwan; 3 Taipei Heart Institute, Taipei Medical University, Taipei 11031, Taiwan; 4 Department of Biomedical Engineering, National Defense Medical Center, Taipei 11490, Taiwan; yaochang.chen@gmail.com; 5 Graduate Institute of Clinical Medicine, College of Medicine, Taipei Medical University, Taipei 11031, Taiwan; 6 Department of Medical Education and Research, Wan Fang Hospital, Taipei Medical University, Taipei 11696, Taiwan; 7 Division of Cardiology, Chang Gung Memorial Hospital, Taoyuan 33305, Taiwan; yeongshinn@cgmh.org.tw; 8 College of Medicine, Chang Gung University, Taoyuan 33302, Taiwan

**Keywords:** fibroblasts, heart failure, left atrium, right atrium, Ca^2+^, CaMKII

## Abstract

Background: Atrial fibrosis plays an important role in the genesis of heart failure and atrial fibrillation. The left atrium (LA) exhibits a higher level of fibrosis than the right atrium (RA) in heart failure and atrial arrhythmia. However, the mechanism for the high fibrogenic potential of the LA fibroblasts remains unclear. Calcium (Ca^2+^) signaling contributes to the pro-fibrotic activities of fibroblasts. This study investigated whether differences in Ca^2+^ homeostasis contribute to differential fibrogenesis in LA and RA fibroblasts. Methods: Ca^2+^ imaging, a patch clamp assay and Western blotting were performed in isolated rat LA and RA fibroblasts. Results: The LA fibroblasts exhibited a higher Ca^2+^ entry and gadolinium-sensitive current compared with the RA fibroblasts. The LA fibroblasts exhibited greater pro-collagen type I, type III, phosphorylated Ca^2+^/calmodulin-dependent protein kinase II (CaMKII), phosphorylated phospholipase C (PLC), stromal interaction molecule 1 (STIM1) and transient receptor potential canonical (TRPC) 3 protein expression compared with RA fibroblasts. In the presence of 1 mmol/L ethylene glycol tetra-acetic acid (EGTA, Ca^2+^ chelator), the LA fibroblasts had similar pro-collagen type I, type III and phosphorylated CaMKII expression compared with RA fibroblasts. Moreover, in the presence of KN93 (a CaMKII inhibitor, 10 μmol/L), the LA fibroblasts had similar pro-collagen type I and type III compared with RA fibroblasts. Conclusion: The discrepancy of phosphorylated PLC signaling and gadolinium-sensitive Ca^2+^ channels in LA and RA fibroblasts induces different levels of Ca^2+^ influx, phosphorylated CaMKII expression and collagen production.

## 1. Introduction

Atrial fibrosis contributes to the genesis of atrial arrhythmia and is the main manifestation of most cardiovascular diseases [1]. A higher level of atrial fibrosis was reported in patients with atrial myopathy or atrial arrhythmia or heart failure (HF) [1,2]. Left atrium (LA) fibrosis is a predictor in patients with atrial fibrillation (AF) [3]. The treatment of atrial fibrosis has been proven to decrease new-onset AF [4]. The LA and right atrium (RA) develop from different embryonic origins and exhibit dissimilar patterns of gene expression with different histological and immunohistochemical properties, which provide multiple clues and targets for disease management [5,6]. The LA exhibits more advanced fibrosis than the RA in patients with atrial arrhythmia [7]. Compared with the RA, the LA exhibited a higher level of pro-fibrotic transcription factors and metalloproteinases in response to angiotensin II treatment [8,9,10]. The gene expression of *Smad 6*, which is an inhibitory messenger of pro-fibrotic cytokine signaling, is more highly expressed in RA than in LA tissues [11,12]. Our previous study revealed a higher collagen production in isolated rat LA fibroblasts than in their RA counterparts. Moreover, the LA had a higher level of fibrosis than the RA in rats with HF [13]. However, the underlying mechanism of the diverse response to pro-fibrotic signaling between LA and RA fibroblasts has not been fully elucidated.

Calcium (Ca^2+^) signaling has been demonstrated to be downstream of multiple pro-fibrotic cytokines [14,15]. Human LA tissue exhibits higher *Pitx2c* expression as compared with RA tissue [16]. *Pitx2c* knockdown induces higher Ca^2+^ influx and fibroblast activity in human atrial fibroblasts [17]. Soluble guanylyl cyclase, a messenger that can decrease collagen production and Ca^2+^ entry, is highly expressed in RA tissues but markedly reduced or absent in LA tissues [18,19,20]. Compared with RA fibroblasts, LA fibroblasts produce a higher level of oxidative stress, which has been found to induce Ca^2+^ entry [13,21]. Increased intracellular Ca^2+^ is mainly derived either from extracellular Ca^2+^ entry or from endoplasmic reticulum (ER) Ca^2+^ release. Extracellular Ca^2+^ can enter cells through (1) voltage-operated Ca^2+^ channels, (2) transient receptor potential (TRP) channels and (3) Orai channels. Patients with AF had a higher expression of TRP channels. In addition, pro-fibrotic cytokines exert their pro-fibrotic effects on atrial fibroblasts through the activation of the TRP channels [22]. TRP canonical (TRPC) 3 and TRPC6 channel-induced Ca^2+^ influx makes a significant contribution to the pathogenesis of fibrosis [23,24]. TRPC3 and TRPC6 are mainly activated by phosphorylated phospholipase C (PLC)-induced diacylglycerol (DAG) [25,26]. PLC also activates inositol trisphosphate (IP3) signaling, inducing Ca^2+^ release from the endoplasmic reticulum (ER) [27,28].

The emptying of Ca^2+^ from the ER can be sensed by a stromal interaction molecule (STIM), a single-pass membrane protein in the ER membrane, thereby activating the store-operated Ca^2+^ entry [29]. Two kinds of STIM (STIM1, STIM2) were discovered in 2000–2001 [30,31]. Sensitized STIM1 clusters transform their conformation and are dominantly conjugated with the surface Orai protein, thereby inducing Ca^2+^ release-activated Ca^2+^ currents [32]. STIM1 has been found to play an important role in cardiac fibrogenesis [33]. STIM2 shares a 61% homology with STIM1 but exhibits different affinities with Ca^2+^ compared with STIM1 and acts as a regulator of the basal intracellular Ca^2+^ level [32,34,35]. The role of STIM2 on cardiac fibrogenesis has not been fully elucidated. However, the diversity of Ca^2+^ entry and the function and expression levels of these Ca^2+^ channels and the regulatory protein between LA and RA fibroblasts have not been fully elucidated. Increasing the Ca^2+^ influx enhances the collagen production capability of fibroblasts [36]. The purpose of the current study was to clarify whether Ca^2+^ signaling contributes to the differences in the collagen production capability between LA and RA fibroblasts.

In the present study, we investigated the Ca^2+^ influx and the membrane Ca^2+^ currents of LA and RA fibroblasts. We evaluated the role of extracellular Ca^2+^ influx and the downstream messenger on the diversity of pro-fibrotic cellular activities and compared the expressions of Ca^2+^ channels and regulatory proteins between LA and RA fibroblasts.

## 2. Materials and Methods

### 2.1. Isolation of LA and RA Cardiac Fibroblasts from Healthy Rats

The study was approved on 8 May 2018 by Laboratory Animal Committee of Taipei Medical University (approval number: LAC-2017-0383). LA and RA cardiac fibroblasts were isolated from male Sprague–Dawley (SD) rats (weighing 300–350 g) by using a modified protocol [13]. Briefly, after the animals were euthanized, the hearts were rapidly mounted on a Langendorff apparatus and perfused with phosphate-buffered saline containing 25 U/mL type 2 collagenase (Sigma, St. Louis, MO, USA) at 37 °C for 35 min. LA and RA tissues were chopped and shaken in phosphate-buffered saline until single fibroblasts were obtained. The cells were filtered through a 40 μm cell strainer and then centrifuged at 300× *g* for 10 min. Isolated atrial fibroblasts were cultured in 6 cm dishes in Dulbecco’s modified Eagle’s medium (Gibco, Paisley, UK) supplemented with 10% fetal bovine serum (Hyclone, Logan, UT, USA) and 100 U/mL penicillin-streptomycin (Gibco). After removing the pre-seeding medium containing the cardiomyocytes, the cells were incubated at 37 °C in the presence of 5% CO_2_ for 48 h and were designated as passage 0 (P0) atrial fibroblasts. The cells were grown to confluence and sub-cultured to passage 1 (P1). P0 and P1 cells were positive for vimentin but negative for CD31 under immunofluorescence microscopy. α-smooth muscle actin was expressed in P1 cells but less in P0 cells (Supplementary Figure S1). P1 atrial fibroblasts, seeded at a density of 3 × 10^5^ cells/cm^2^ on culture dishes, were used in subsequent experiments and incubated in a serum-free medium for 24 h before each assay. P1 cells were used for Western blotting as more cells were required for the experiments.

### 2.2. Intracellular Ca^2+^ Imaging

Ca^2+^ imaging was performed as described previously [37]. The P0 atrial fibroblasts on 3 cm glass-bottom chamber slides were loaded with fura-2-acetoxymethyl ester (5 μmol/L; Invitrogen, Carlsbad, CA, USA) and Pluronic F-127 (20% solution in DMSO, 2.5 μg/mL) in a Ca^2+^-free solution containing (in mmol/L) NaCl 120, KCl 5.4, KH_2_PO_4_ 1.2, MgSO_4_ 1.2, glucose 10, HEPES 6 and Taurine 8 (pH 7.40) for 30 min at 36 °C in a humidified incubator with 5% CO_2_. Fura-2 fluorescence images were acquired using a Polychrome V (Till Photonics, Munich, Germany) monochromator mounted on an upright Leica DMI 3000B microscope with dual excitation wavelengths of 340 and 380 nm and an emission wavelength of 510 nm. The fura-2 images were analyzed using MetaFluor software version 7.7.6.0 (Molecular Devices, Sunnyvale, CA, USA). The ratio of F_340_ to F_380_ was used as a marker for the relative level of intracellular Ca^2+^. To measure Ca^2+^ entry, the cells were first exposed to the Ca^2+^-free solution for 8 min. The extracellular Ca^2+^ concentration was then increased to 10 mmol/L to measure Ca^2+^ entry through the store-operated channels activated by the Ca^2+^-store depletion. The intracellular Ca^2+^ was measured from the average of F_340_/F_380_ during 300–400 s under an extracellular-free Ca^2+^ solution (F_340_/F_380_-free Ca^2+^). The final peak of intracellular Ca^2+^ was measured from the average of F_340_/F_380_ during 2300–2400 s under 10 mmol/L Ca^2+^ solution (F_340_/F_380_ 10 mmol/L Ca^2+^). The change (∆ F_340_/F_380_) between (F_340_/F_380_-free Ca^2+^) and (F_340_/F_380_ 10 mmol/L Ca^2+^) was used to represent the Ca^2+^ entry of each cell.

### 2.3. Patch Clamp Experiments

Subsequent to a gigaseal (seal resistance between 1–4 GΩ), a whole-cell patch clamp was performed on detached single P0 fibroblasts using an Axopatch 1D amplifier (Axon Instruments, Foster City, CA, USA) as described previously [24]. The area under the capacitive current was activated using a small hyperpolarizing step from a holding potential of −50 mV to a test potential of −55 mV for 80 milliseconds. The measured membrane resistance for P0 atrial fibroblasts was 0.49 ± 0.05 GΩ. When measuring gadolinium-sensitive currents, the detached fibroblasts were superfused with a Tyrode solution containing (mmol/L): NaCl 140, TEA-Cl 5.4, MgCl 1.0, CaCl_2_ 2.0, glucose 10 and HEPES 10 with pH: 7.4 adjusted using CsOH. The pipette solution contained (mmol/L): CsCl_2_ 135, CaCl_2_ 0.1, EGTA 10, Mg-ATP 4.0, MgCl_2_ 1.0, HEPES 10, Na-GTP 0.3, Na_2_-phosphocreatine 6.6 with pH: 7.4 adjusted with CsOH. The currents were the differences before and after gadolinium (100 μmol/L, Sigma-Aldrich, St. Louis, MO, USA) recorded by a voltage ramps for 3 s ranging from −110 mV to +100 mV (0.07 mV/ms, 0.1 Hz) at 37 °C. Nifedipine (5 μmol/L) was used in the external solution to block any l-type Ca^2+^ current.

### 2.4. Western Blotting

Western blotting was performed as described previously [13]. In brief, P1 LA and RA fibroblasts treated with or without ethylene glycol tetra-acetic acid (EGTA, 1 mmol/L, Sigma-Aldrich) or a Ca^2+^/calmodulin-dependent protein kinase II (CaMKII) inhibitor (KN93, 10 μmol/L, Sigma-Aldrich) were lysed in a radioimmunoprecipitation assay buffer containing 150 mmol/L NaCl, 0.5% sodium deoxycholate, 1% NP40, 50 mmol/L Tris pH 7.4, 0.1% sodium dodecyl sulfate (SDS) and protease inhibitor cocktails (Sigma). The proteins were fractionated using 10% SDS-polyacrylamide gel electrophoresis and transferred onto an equilibrated polyvinylidene difluoride membrane (Amersham Biosciences, Buckinghamshire, UK). The membranes were then incubated with primary antibodies against pro-collagen type III (1:3000, monoclonal, clone number: FH-7A, Abcam, Cambridge, UK); pro-collagen type IA1 (1:500, monoclonal, clone number: 3G3, Santa-Cruz Biotechnology, Santa Cruz, CA, USA); PLC-γ1 (1:500, polyclonal, Cell Signaling Technology, Beverly, MA, USA); STIM1 (1:2000, monoclonal, clone number: 44, BD Transduction Laboratories, San Diego, CA, USA); phosphorylated CaMKII (1:3000, polyclonal, Abcam); Orai (1:2000, polyclonal, PROSCI, Poway, CA, USA); TRPC3 (1:1000, polyclonal, Abcam); and TRPC6 (1:3000, polyclonal, Alomone Labs, Jerusalem, Israel) and secondary antibodies. The bound antibodies were visualized using an ECL detection system (Millipore, Darmstadt, Germany) and analyzed with AlphaEaseFC software version 4.0.0 (Alpha Innotech, San Leandro, CA, USA). GAPDH (Sigma) was used as the loading control and normalized to the value of LA control fibroblasts.

### 2.5. Induction of Heart Failure

The induction of heart failure was performed as described previously [13]. Whole HF was induced in male Sprague–Dawley rats (weighing 300–350 g) by a subcutaneous injection of a high dose of isoproterenol (100 mg/kg). HF rats were euthanized 12 days after the isoproterenol injection for HF LA and RA fibroblast isolation.

### 2.6. Statistical Analysis

All quantitative data are expressed as a mean ± standard error of the mean. The differences between LA and RA cardiac fibroblasts were compared using the unpaired *t*-test, paired *t*-test, Mann–Whitney rank-sum test or Wilcoxon signed-rank test depending on the outcome of the normality test. The differences between the different groups were compared by a two-way repeated ANOVA test with a post hoc of a Fisher LSD test. A *p* value of <0.05 was considered statistically significant.

## 3. Results

### 3.1. Diversity in Ca^2+^ Entry Between Isolated P0 LA and RA Fibroblasts from Healthy Rats

**Figure 1:**
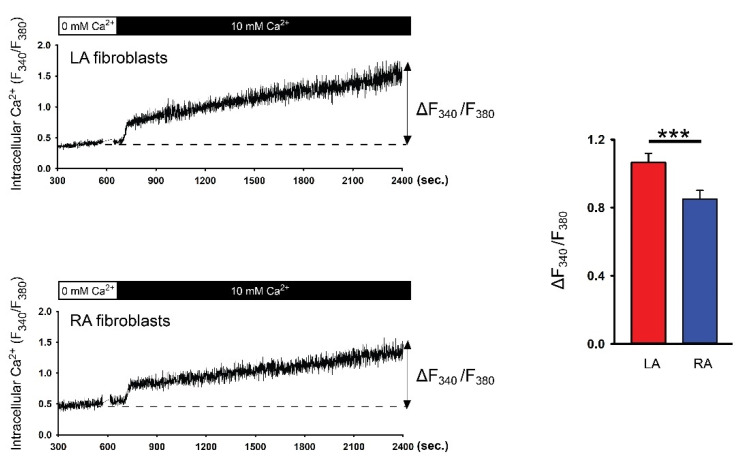
Ca^2+^ entry in the isolated passage 0 (P0) left atrium (LA) and right atrium (RA) fibroblasts from healthy rats. Left panels represent intracellular Ca^2+^ tracing from the LA fibroblasts (upper tracing, n = 18 LA fibroblasts from four rats) and RA fibroblasts (lower tracing, n = 17 RA fibroblasts from four rats). The cells were first incubated with a Ca^2+^-free extracellular solution to deplete the Ca^2+^ stores. Ca^2+^ entry was induced after increasing extracellular Ca^2+^ to 10 mmol/L. Right panels show the change in intracellular Ca^2+^ from a Ca^2+^-free solution to a 10 mmol/L Ca^2+^ solution (∆F_340_/F_380_). *** *p* < 0.005.

**Figure 2:**
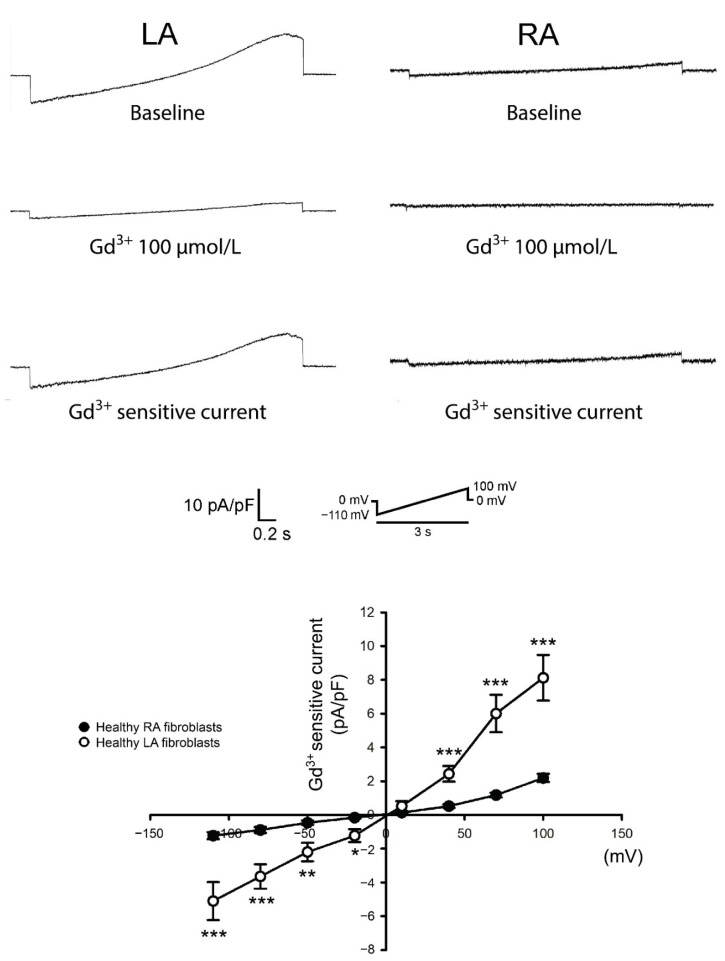
Membrane gadolinium (Gd^3+^)-sensitive currents in isolated passage 0 (P0) left atrium (LA) and right atrium (RA) fibroblasts from healthy rats. Left panels reveal tracings of the Gd^3+^ (100 μmol/L)-sensitive non-selective cation current from LA fibroblasts (n = 10 from five rats) and RA fibroblasts (n = 10 from five rats). Right panels reveal the current/voltage (I/V) relationship of the Gd^3+^-sensitive non-selective cation current. * *p* < 0.05, ** *p* < 0.01, *** *p* < 0.005. The insets in the current traces show the various clamp protocols.

To evaluate the Ca^2+^ entry diversity between LA and RA P0 fibroblasts, these cells were first incubated with a Ca^2+^-free extracellular solution to deplete the Ca^2+^ stores. Ca^2+^ entry was induced after increasing extracellular Ca^2+^ to 10 mmol/L. A fura-2 fluorescence image revealed that P0 LA fibroblasts exhibited a higher Ca^2+^ entry compared with P0 RA fibroblasts in healthy rats (Figure 1). Gadolinium has been considered as a non-specific TRP channel inhibitor [24]. We evaluated a gadolinium-sensitive cation current in the patch clamp experiments to study the role of the TRP channels in the diversity of Ca^2+^ entry between LA and RA fibroblasts. We found that P0 LA fibroblasts from healthy rats showed higher gadolinium-sensitive currents compared with P0 RA fibroblasts (Figure 2).

### 3.2. Differences in Ca^2+^ Signaling between Cultured P1 LA and RA Fibroblasts from Healthy Rats

**Figure 3:**
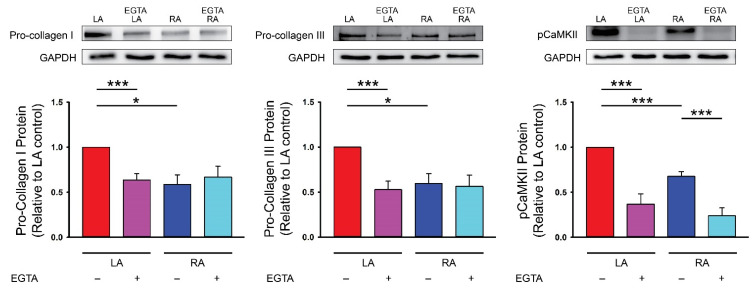
Ca^2+^ entry on the diversity of the collagen production ability in the cultured passage 1 (P1) left atrium (LA) and right atrium (RA) fibroblasts from healthy rats. Photographs and averaged data of the pro-collagen type I (n = 6 independent experiments), pro-collagen type III (n = 6 independent experiments) and phosphorylated Ca^2+^/calmodulin-dependent protein kinase II (pCaMKII, n = 6 independent experiments) expression in LA and RA fibroblasts with or without 1 mmol/L ethylene glycol tetra-acetic acid (EGTA, an extracellular Ca^2+^ chelator) for 48 h. GAPDH was used as a loading control. * *p* < 0.05, *** *p* < 0.005.

**Figure 4:**
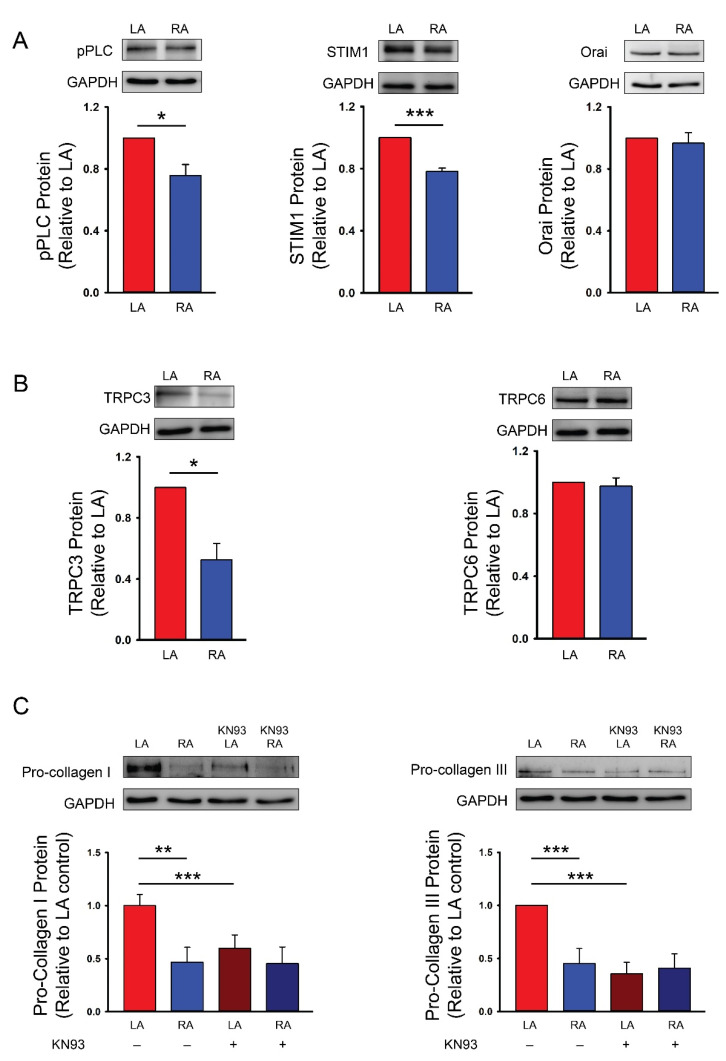
Ca^2+^ signaling pathway on the diversity of the collagen production capability between the cultured passage 1 (P1) left atrial (LA) and right atrial (RA) fibroblasts from healthy rats. (**A**) Photographs and averaged data of the phosphorylated phospholipase C (pPLC, n = 5 independent experiments), stromal interaction molecule 1 (STIM1, n = 5 independent experiments) and Orai (n = 5 independent experiments) expression of LA and RA fibroblasts. (**B**) Photographs and averaged data of the transient receptor potential canonical (TRPC) 3 (TRPC3, n = 5 independent experiments) and TRPC6 (n = 5 independent experiments) expression of LA and RA fibroblasts. (**C**) Photographs and averaged data of the pro-collagen type I (n = 5 independent experiments) and pro-collagen type III (n = 5 independent experiments) expression of LA and RA fibroblasts with or without a Ca^2+^/calmodulin-dependent protein kinase II (CaMKII) inhibitor (KN93, 10 μmol/L) for 48 h. GAPDH was used as a loading control. * *p* < 0.05, ** *p* < 0.01, *** *p* < 0.005.

LA fibroblasts exhibited higher pro-collagen type I, type III and phosphorylated CaMKII expressions compared with RA fibroblasts. EGTA is an extracellular Ca^2+^ chelator and has been used to evaluate the role of extracellular Ca^2+^ on Ca^2+^ entry in various cellular activities [38]. In the present study, EGTA (1 mmol/L) treatment reduced pro-collagen type I, type III and phosphorylated CaMKII expressions in LA fibroblasts. EGTA treatment reduced phosphorylated CaMKII but not the pro-collagen type I and type III expressions in RA fibroblasts. Furthermore, in the presence of EGTA (1 mmol/L), LA and RA fibroblasts had similar pro-collagen type I, type III and phosphorylated CaMKII expressions suggesting that differential pro-fibrotic activity between the primary isolated LA and RA fibroblasts was endogenously regulated by Ca^2+^ entry (Figure 3). We studied the molecular expression of PLC in LA and RA fibroblasts and found that LA fibroblasts had a greater phosphorylated PLC level (Figure 4). Moreover, LA fibroblasts also showed a greater STIM1 expression suggesting that LA fibroblasts may have upregulated the store-operated Ca^2+^ entry compared with RA fibroblasts (Figure 4). To evaluate the downstream signaling of PLC, we studied the expression levels of TRPC3 and TRPC6 and found that LA fibroblasts expressed a higher TRPC3 compared with RA fibroblasts. However, LA and RA fibroblasts exhibited similar levels of Orai and TRPC6 expressions (Figure 4).

We used KN93 (10 μmol/L, a CaMKII inhibitor) to evaluate the role of higher phosphorylated CaMKII in the LA fibroblasts and found that the LA and RA fibroblasts exhibited similar pro-collagen type I and type III levels in the presence of KN93 (Figure 4) suggesting that CaMKII phosphorylation regulated the diversity of the collagen production between LA and RA fibroblasts.

### 3.3. Differences in Gadolinium-Sensitive Currents between Isolated P0 LA and RA Fibroblasts from HF Rats

**Figure 5:**
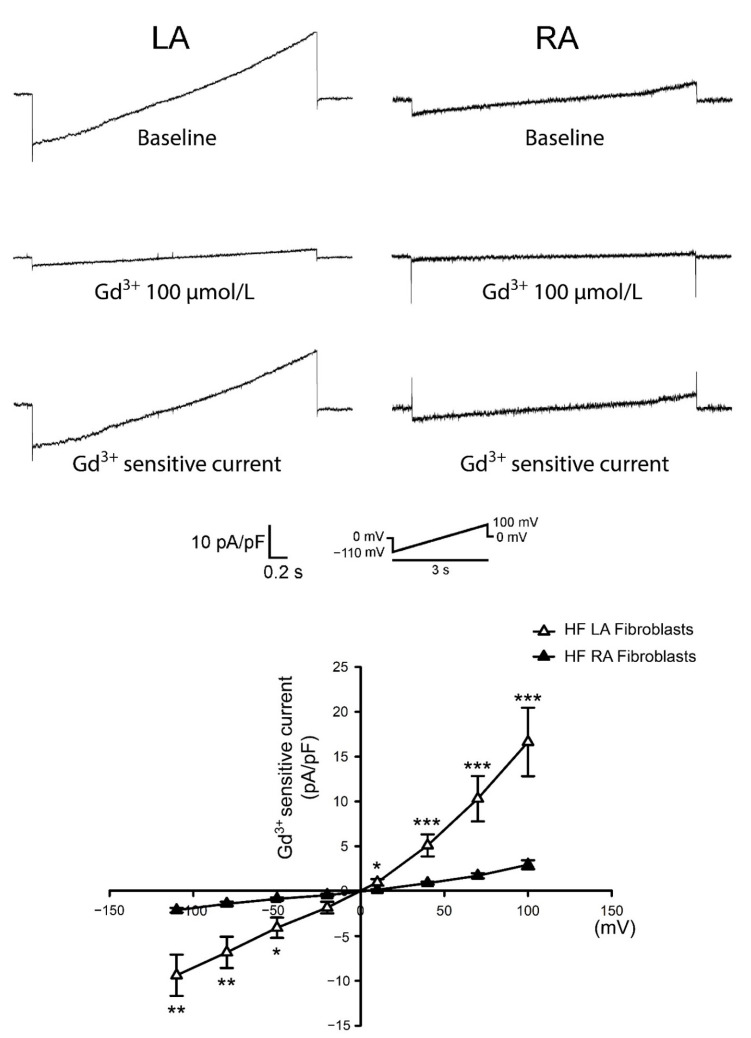
Membrane gadolinium (Gd^3+^)-sensitive currents in isolated passage 0 (P0) left atrium (LA) and right atrium (RA) fibroblasts from heart failure (HF) rats. Left panels reveal tracings of the Gd^3+^ (100 μmol/L)-sensitive non-selective cation current from HF LA fibroblasts (n = 10 from six rats), HF RA fibroblasts (n = 10 from five rats). Right panels reveal the current/voltage (I/V) relationship of the Gd^3+^-sensitive non-selective cation current. * *p* < 0.05, ** *p* < 0.01, *** *p* < 0.005. The insets in the current traces show the various clamp protocols.

In our previous study [13], we found that in HF rats, LA tissues expressed higher fibrotic levels compared with RA tissues. We evaluated the diversity of Ca^2+^ homeostasis between HF LA and HF RA fibroblasts. We found that P0 LA fibroblasts from HF rats showed higher gadolinium-sensitive currents compared with P0 RA fibroblasts (Figure 5). Moreover, compared to healthy P0 LA fibroblasts, HF P0 LA fibroblasts exhibited higher gadolinium-sensitive currents (Supplementary Figure S2), which was comparable with the findings of previous studies that HF fibroblasts exhibited upregulated currents through TRP channels compared with fibroblasts from healthy subjects [24,39]. However, HF RA and healthy RA fibroblasts had similar gadolinium-sensitive currents.

## 4. Discussion

A higher degree of fibrosis in the LA than the RA has been found in various cardiovascular diseases [13,40]. The Ca^2+^ signaling pathway plays an important role in pro-fibrotic cellular activities. However, whether Ca^2+^ homeostasis contributes to the diversity between LA and RA fibroblasts remains unclear. Here we have shown, for the first time, that a difference in the Ca^2+^ signaling induced the collagen production between LA and RA fibroblasts. We found that the Ca^2+^ entry capability was greater in isolated rat LA fibroblasts than in their RA counterparts from healthy rats. In addition, EGTA-treated LA and RA fibroblasts exhibited a similar collagen production ability, indicating that the Ca^2+^ influx activated the augmented pro-fibrotic activities in LA fibroblasts. Ca^2+^ influx has been the target of treatment for various fibrotic diseases. Fibroblasts isolated from patients with systemic sclerosis exerted their activated pro-fibrotic cellular activities through a Ca^2+^ influx [41]. Transforming growth factor (TGF)-β augmented collagen production through promoting Ca^2+^ entry whereas EGTA attenuated TGF-β-mediated collagen production in renal fibroblasts [42]. A platelet-derived growth factor (PDGF) evoked a Ca^2+^ influx thereby inducing the collagen production of lung fibroblasts [15]. Compared with RA tissue, LA tissue exhibited higher levels of calcitonin gene-related peptide, which is a cardiovascular neurotransmitter that can increase the intracellular Ca^2+^ amount through Ca^2+^ entry [43,44]. In addition, a pro-fibrotic protease chymase, which is highly expressed in LA tissues but not in RA tissues, can induce Ca^2+^ entry [45,46,47]. Accordingly, LA fibroblasts may constitutionally exhibit a higher Ca^2+^ influx capability compared with RA fibroblasts.

Pro-fibrotic cytokines such as TGF-β or PDGF induce an extracellular matrix production through the PLC signaling pathway [15,48]. The inhibition of phospholipase C signaling can decrease the collagen production capabilities of lung fibroblasts [49]. Studies have evaluated the differences between the LA and the RA. A genomic study revealed that the gene expression in LA tissue had a greater involvement in Wnt signaling compared with RA tissue in patients with AF [50]. The non-canonical Wnt signaling pathway activates phosphorylated PLC, thereby increasing IP3 production and inducing Ca^2+^ homeostasis [51]. The gene expression of aldose reductase, which is a protein that can activate the PLC signaling pathway and enhance Ca^2+^ influx, is more highly expressed in LA than in RA tissues [11,52,53]. Moreover, the activation of the Notch signaling pathway was found in the LA but not in the RA in patients with atrial arrhythmia [54]. Notch activation can upregulate STIM1 expression and activate store-operated Ca^2+^ entry [55,56]. The present study showed that LA fibroblasts expressed a higher level of phosphorylated PLC and STIM1 compared with RA fibroblasts. Our findings suggested a high propensity for Ca^2+^ homeostasis in LA fibroblasts, which might contribute to a higher fibrogenesis in LA fibroblasts compared with their RA counterparts. The expression of the STIM1/Orai conjugation was positively correlated with a greater collagen production capability in cardiac fibroblasts [57]. The different levels of STIM1 in LA and RA fibroblasts may also contribute to the diversity of collagen production via the activation of the Orai channel. However, as we studied atrial fibrogenesis using healthy cells taken from healthy tissues it is not clear whether these findings can be translated to pathological conditions.

We found that the gadolinium-sensitive current was higher in LA fibroblasts than RA fibroblasts isolated from healthy rats. Compared with RA fibroblasts, LA fibroblasts exhibited higher expression levels of TRPC3 but not TRPC6. Genetically knocking out TRPC3 attenuated myocardial fibrosis in pressure-overloaded HF mice [58]. Our findings suggested that the higher gadolinium-sensitive current in LA fibroblasts might be related to the TPRC3 channel. The diversities in the electrophysiologic characteristics between LA and RA tissues have been studied [59]. LA tissues exhibited a higher inward-rectifier potassium channel gene expression compared with RA tissues [60]. Differences in electrophysiologic currents have been found between the LA and left ventricular fibroblasts [61]. Moreover, we found that in rats with HF, isolated LA fibroblasts also exhibited higher gadolinium-sensitive currents compared with isolated RA fibroblasts. Accordingly, we may speculate that, compared with RA fibroblasts, LA fibroblasts exhibited more currents through TRPC3, thereby exhibiting a greater collagen production capability. However, gadolinium has been proven to inhibit currents through Orai channels [62,63]. Gadolinium-sensitive currents could also be the result of STIM-Orai-dependent store-operated calcium entry.

**Figure 6:**
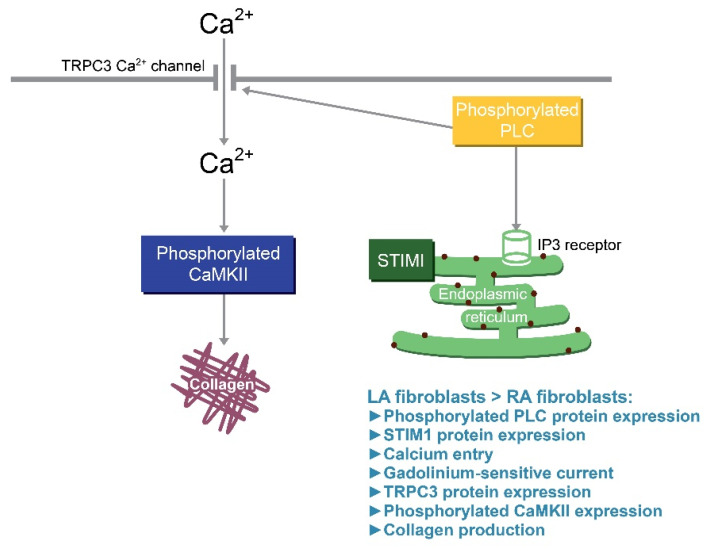
Illustration of the proposed mechanism that contributes to the differential collagen production in left atrium (LA) and right atrium (RA) fibroblasts. The diversities of the phosphorylated phospholipase C (pPLC) signaling and the expression of transient receptor potential canonical (TRPC) 3 Ca^2+^ channels activate different levels of Ca^2+^ influx, which may induce a different phosphorylated Ca^2+^/calmodulin-dependent protein kinase II (CaMKII) expression and collagen production between LA and RA fibroblasts. The different levels of stromal interaction molecule 1(STIM1) in LA and RA fibroblasts may also contribute to the diversity of collagen production via the activation of the Orai channel. IP3: Inositol trisphosphate.

CaMKII, triggered by Ca^2+^/calmodulin-induced auto-phosphorylation, is a downstream messenger of the Ca^2+^ signaling pathway [64]. CaMKII activation contributes to pathological cardiac remodeling as the expression is upregulated in patients with AF or HF [65,66]. CaMKII activation augments the collagen production in cardiac fibroblasts [67]. Moreover, genetically knocking-down CaMKII or a treatment with KN93 was shown to reduce myocardial fibrosis in mice with pathological remodeling [68,69]. KN93 can also inhibit the proliferation, collagen production and pro-fibrotic cytokine production capability of cardiac fibroblasts [67]. Our study showed that EGTA-treated LA and RA fibroblasts had similar levels of phosphorylated CaMKII. LA and RA fibroblasts had a similar collagen production capability upon KN93 treatment suggesting that a higher Ca^2+^ influx induced the CaMKII signaling pathway further, thereby activating the augmented pro-fibrotic cellular activity of LA fibroblasts. Figure 6 shows the proposed mechanism that contributed to the differential collagen production in LA and RA fibroblasts. The diversities of phosphorylated PLC signaling and the expression of TRPC3 and STIM1 activated different levels of Ca^2+^ influx resulting in a different phosphorylated CaMKII expression and collagen production between LA and RA fibroblasts.

There were a few limitations in this study. Although gadolinium has been used as a non-specific TRP channel blocker for years [70,71], gadolinium can also indirectly inhibit ATP-gated P2X receptor cation (P2X) or chloride channels [72,73]. Hence, it is not clear whether P2X or chloride channels contributed to the diversity of the Ca^2+^ influx [74,75]. In addition, this study found a different constitutive discrepancy in the Ca^2+^ currents between LA and RA fibroblasts but the displayed currents were unspecific due to no activation stimulus (receptor stimulation, direct agonists, etc.) being applied. Moreover, the cells were incubated in a nominal calcium-free solution [24], which might be insufficient to induce a proper calcium store depletion. The addition of EGTA to the extracellular solution and the inhibition of the SERCA pumps with cyclopiazonic acid or thapsigargin is regularly used to empty calcium stores. However, this study did not use this method to avoid ER stress-induced fibroblast death [76] as the cells were primarily isolated and invulnerable to this treatment. Therefore, our findings might underestimate the differences of LA and RA fibroblasts on ER Ca^2+^ release. Finally, part of the diversity of the Ca^2+^ level at the end of Ca^2+^ imaging might be also due to the differences in the cytosolic calcium clearance between LA and RA fibroblasts as our study did not clarify the role of the SERCA pump and plasma membrane Ca^2+^ ATPase (PMCA) pump in the Ca^2+^ imaging.

In conclusion, the discrepancy of phosphorylated PLC signaling and gadolinium-sensitive Ca^2+^ channels in LA and RA fibroblasts induced different levels of Ca^2+^ influx, phosphorylated CaMKII expression and collagen production.

## Data Availability

The data that support the findings of this study are available from the corresponding author upon reasonable request.

## Supplementary Materials

The following are available online at https://www.mdpi.com/article/10.3390/biomedicines9060686/s1, Figure S1: α-smooth muscle actin (α-SMA) expression in passage 0 (P0) and passage 1 (P1) left atrium (LA) and right atrium (RA) fibroblasts. Figure S2: Current/voltage (I/V) relationship of the membrane gadolinium (Gd3+)-sensitive currents in isolated passage 0 (P0) left atrium (LA) and right atrium (RA) fibroblasts from healthy and heart failure (HF) rats.
