# Supplementary material for: Calcium Regulation on the Atrial Regional Difference of Collagen Production Activity in Atrial Fibrogenesis
Source: Biomedicines. 2021 Jun 17;9(6):686. doi: 10.3390/biomedicines9060686 (PMC8233809; doi:10.3390/biomedicines9060686)
Supplement: Supplementary file 1 [file biomedicines-09-00686-s001.zip › biomedicines-1233082-supplementary.pdf]

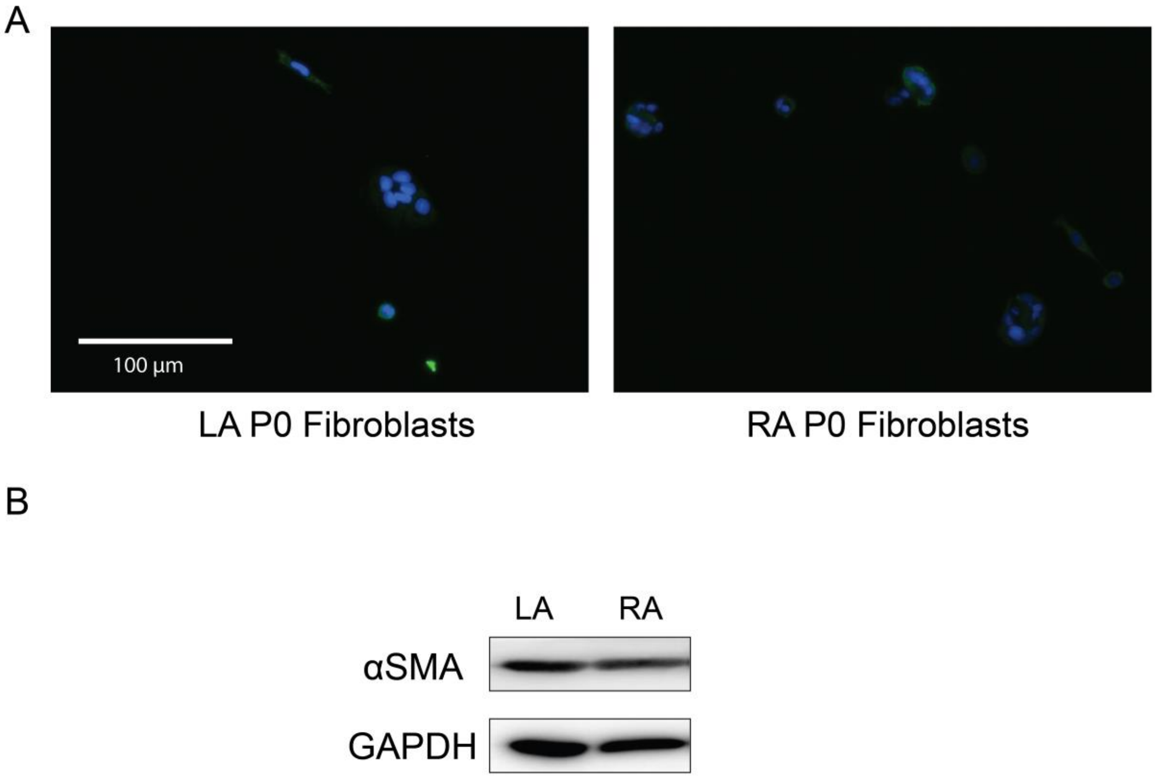

Figure S1. α-smooth muscle actin (α-SMA, marker of fibroblast activation) expression in passage 0 (P0) and passage 1 (P01) left atrium (LA) and right atrium (RA) fibroblasts. (A) Photographs of immunofluorescent staining of α-SMA stained green in P0 LA and RA fibroblasts with DAPI immunofluorescent staining (stained blue). P0 LA and RA fibroblasts expressed weak α-SMA expression. (B) Photographs of the α-SMA protein expression in P1 LA and RA fibroblasts. LA and RA P1 fibroblasts exhibited significant α-SMA expression.

Methods of Supplmentary Figure S1

Immunofluorescence microscopy

Cells were fixed in PBS containing 4% paraformaldehyde for 30 min, blocked with 3% bovine serum albumin followed with anti α-SMA antibody (1:200, monoclonal, clone number: 1A4, Abcam) incubation for 2 h. Cells were then incubated in goat anti-mouse IgG Alexa Fluor 488 (Invitrogen, Carlsbad, CA, USA) at a dilution of 1:200 for 1 h. Then, the cells were mounted in Vectashield (Vector Laboratories, Inc., Burlingame, CA, USA) with 4´,6-diamidino-2- phenylindole for measurement by immunofluorescence microscopy.

Western blotting

The procedure of the western blotting was described in the manuscript. Blots were probed with primary antibodies against α-SMA (1:1000, monoclonal, clone number: 1A4, Abcam).

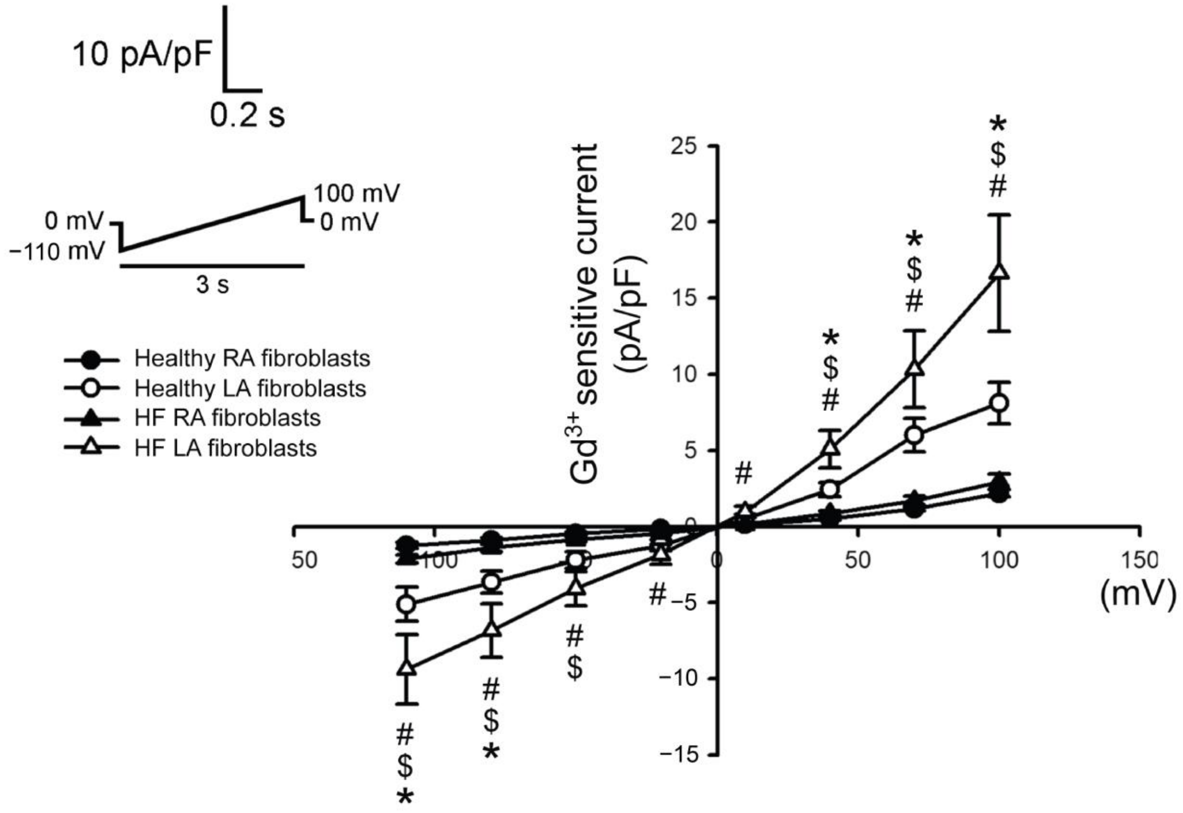

Figure S2. Current/voltage (I/V) relationship of the Membrane gadolinium (Gd^{3+})-sensitive currents in isolated passage 0 left atrium (LA) and right atrium (RA) fibroblasts from healthy and heart failure (HF) rats. HF LA fibbroblasts (n = 10) had larger Gd^{3+} sensitive currents than healthy LA fibroblasts (n = 10). HF RA (n = 10) and healthy RA (n = 10) fibroblasts had similar Gd^{3+} sensitive currents. Differences between different groups were compared by the two-way ANOVA test with a post hoc of Fisher LSD test. The insets in the current traces showed the various clamp protocols. * Healthy LA versus healthy RA fibroblasts; $ HF LA versus healthy LA fibroblasts; # HF LA versus HF RA fibroblasts.
